# Turnover Kinetics of Pancreatic Macrophages in Lean and Obese Diabetic Mice

**DOI:** 10.3389/fendo.2022.858422

**Published:** 2022-07-12

**Authors:** Ziyuan Ma, Christiane Ruedl

**Affiliations:** School of Biological Sciences, Nanyang Technological University, Singapore, Singapore

**Keywords:** pancreas, macrophages, obesity, diabetes, turnover kinetics, replenishment, bone marrow

## Abstract

Pancreatic resident macrophages, a heterogeneous family of cells with distinct origins and phenotypes, are the main myeloid cells in exocrine and endocrine tissues. Adult exocrine F4/80^hi^ macrophages consist of three different subsets based on the embryonic marker Tim-4 and MHC II expression. Their frequencies shift during aging and obesity with the Tim-4^-^MHCII^+^ fraction becoming the predominant subpopulation in the inter acinar stroma. Endocrine resident F4/80^hi^ macrophages are more homogenous and represent the prevalent leukocyte fraction residing within the islets in both lean and obese mice. We used an adult fate mapping mouse model to characterize turnover kinetics within the pancreatic resident macrophages under normal homeostasis and obese diabetic conditions. We demonstrate that islet resident macrophages show unique replenishment kinetics, with embryonic macrophages being gradually replaced by bone marrow-derived monocytes with increasing age. Their replenishment was independent of the CCL2/CCR2 axis. Furthermore, we confirmed that both exocrine Tim-4^+^MHCII^low^ and Tim-4^+^MHCII^+^ fractions are long-lived and primarily independent from bone marrow-derived monocytes. In contrast, exocrine Tim-4^-^MHCII^+^ macrophages are gradually replaced through a CCR2-dependent influx of bone marrow-derived monocytes in aging. Moreover, we show that obesity and type 2 diabetes do not affect the turnover kinetics of any macrophage subpopulation residing in the pancreas. Our study uncovers new insights on pancreatic macrophage biology in aging and obesity.

## Introduction

Tissue-resident macrophages are a heterogeneous family of innate immune cells found in many tissues and organs (1). Distinct macrophage subpopulations display unique tissue-specific functions that are not limited to immune surveillance, tissue homeostasis and tissue repair (2). They also contribute to initiating and propagating diseases like cancer (3, 4), chronic inflammation and metabolic disorders, including insulin resistance and type 2 diabetes mellitus (T2DM) (5).

Most tissue-resident macrophages develop during definitive hematopoiesis from fetal monocytes with few exceptions. In adulthood, they are long-lived, maintaining their numbers through self-renewal. Under certain circumstances, depending on the local tissue microenvironment, they are replenished at different kinetics by bone-marrow (BM)-derived monocytes, which can contribute to the maintenance of the local macrophage pool (6–11).

The pancreas, a dual-function gland with glucose-regulating endocrine and exocrine digestive activity, is seeded by a diverse spectrum of myeloid cells. The acinar stroma harbors a heterogeneous myeloid cell population, including tissue-resident macrophages, monocytes, and dendritic cells. In the smaller endocrine gland, the main population is represented by tissue-resident macrophages, which comprise more than 90% of the CD45^+^ immune cell population in the islets (12). Exocrine and endocrine resident macrophages are different not only in their phenotype but also in their ontogeny and function (12). Macrophages are crucial in the development of the endocrine pancreas and β-cells, since their absence leads to a reduction in β-mass, abnormal islet morphology and defects in β-cell proliferation (13, 14). In addition, there is emerging evidence that macrophages cross-talk with other cells within the pancreas. The involvement of macrophages in pancreas healing and regeneration and in the development and progression of pancreatic ductal adenoma have been recently suggested (15) but still not fully elucidated.

Likewise, the contribution of pancreatic macrophages in Type 1 and 2 diabetes is still controversial [reviewed in (16)] since both detrimental (e.g. β-cell dysfunction and death) (17, 18) and beneficial (e.g. pancreas and β-cell regeneration) (19–21) features have been reported.

To further understand the biology of pancreatic macrophages in this metabolic disorder, resident macrophages within the pancreatic microenvironment were characterized in healthy and obese diabetic mice. We investigated their turnover kinetics and analyzed their dependency on monocytes replenishment using a fate-mapping mouse. We show that intra acinar F4/80^hi^Tim-4^-^MHCII^+^ macrophages are replaced by BM-derived circulating monocytes, whereas the remaining two exocrine F4/80^hi^Tim-4^+^ subpopulations are long-lived and minimally refilled by monocytes. Similarly, islets F4/80^hi^ macrophages are progressively replenished over time by BM-derived monocytes, but this happens in a CCR2-independent manner. Remarkably, chronic conditions associated with enhanced inflammatory activities such as obesity and T2DM, do not accelerate the turnover kinetics of pancreatic macrophages as they do in the adipose tissue.

## Materials and Methods

### Mice and Diet

Kit^*MerCreMer*^/R26^*YFP*^ mice were generated as previously described (9). C57BL/6J, CX3CR1^GFP^ and CCR2^-/-^ mice (B6.129S4-Ccr2tm1Ifc/J) were obtained from The Jackson Laboratory (Bar Harbor, ME, USA). Only male mice were used for all experiments performed. All mice were bred and maintained in a specific pathogen-free animal facility of the Nanyang Technological University (Singapore).

To induce obesity and T2DM, 6-8 week-old mice were fed with a western diet (WD) composed of 40 kcal % of fat and 43 kcal % of carbohydrate (Research Diet, D12079Bi, New Brunswick, NJ, USA). The body weight was monitored on a weekly basis and after 24 weeks, the mice reached on average a bodyweight between 45-50 g and were sacrificed for collection of pancreas.

### Tamoxifen-Inducible Adult Fate-Mapping Mouse Model

Kit^*MerCreMer*^/R26^*YFP*^ fate-mapping mice were used to trace the ontogeny and turnover rates of distinct myeloid cell subsets. A total of 4 mg tamoxifen (TAM) (T5648; Sigma-Aldrich, St. Louis, MO, USA) per mouse was administered for five consecutive days by oral gavage for adult labelling as previously described (9). After TAM administration, c-kit^+^ hematopoietic stem cells (HSCs)/progenitors localized in the bone marrow (BM) of Kit^*MerCreMer*^/R26^*YFP*^ mice were labelled with yellow fluorescent protein (YFP^+^) and all cells deriving from YFP^+^ BM-HSC/progenitors would maintain YFP expression when seeding the peripheral tissues/organs. This effect makes it possible to delineate macrophages that are derived from adult BM-definitive haematopoiesis.

### Macrophage Isolation From Islets and Pancreatic Exocrine Stroma

The pancreas was perfused through the bile duct with 250 μg/ml collagenase P (Sigma Aldrich, St. Louis, MO, USA) diluted in HBSS (Sigma Aldrich). After perfusion the pancreata were removed, cut into small pieces digested with collagenase P at 37°C for 15 minutes. After vigorous shaking for 1 min, the homogenous mixture was passed through a metal sieve and larger chunks were further broken down through grinding. Subsequently, the mixture was poured through a 70 μm cell filter allowing exocrine cells passing through, whereas islets remained retained in the strainer. The islets were transferred into Petri dishes, handpicked under a microscope and incubated 10 min at 37°C with Accutase^®^ cell detachment solution (Biolegend, San Diego, CA, USA) to obtain a single cell solution. The exocrine pancreas was separately incubated with DNase I (20 U/ml) (Life Technologies, Carlsbad, CA, USA) at 37°C for 1 hour. The obtained cells were passed through a 100 μm filter and resuspended in NH_4_CL red cell lysis buffer for 5 min to eliminate contaminating erythrocytes.

### Flow Cytometry Staining

Endocrine and exocrine pancreatic single-cells were pre-incubated with 10 μg/ml anti-Fc receptor antibody for 15 min on ice at 4°C in the dark, further incubated with fluorochrome-labelled antibodies at 4°C for another 20 min, and then washed and re-suspended in PBS 2% for analysis on five-laser flow cytometers (FACSymphony™; BD Bioscience, San Jose, CA, USA). The following antibodies were used: APC-Cy7-labelled CD45 (30F11), BV605-labelled Ly6C (HK1.4), BV510-labelled CD11c (N418), Alexa Fluor700-labelled CD206 (C068C2), Percp-Cy5.5-labelled CD301 (LOM-14), APC-Cy7-labelled CD11b (M1/70), Pe-Cy7 and Alexa Fluor647-labelled Tim-4 (RMT4-54) all purchased from Biolegend. PE-labelled F4/80 (BM8), eFluor450-labelled MHC class II (M5/114.15.2) and PE-Cy7-labelled CD11c (N418) all purchased from ThermoFisher Scientific (Waltham, MA, USA). Percp-Cy5.5-labelled CD11b (M1/70) was purchased from BD Biosciences and APC-labelled anti-CCR2 was purchased from R&D Systems (Minneapolis, NM, USA).

### Analysis of β-Islet Size and Islet Macrophage Numbers

The pancreas was fixed in 10% formalin at autopsy followed by dehydration in 30% sucrose and then embedded in Optimal Cutting Temperature (O.C.T.) compound (Tissue Tek, Sakura Finetek, Torrance, CA, USA) for subsequent analysis. ten μm sections were cut and stained with anti-mouse insulin antibody (Cell Signaling Technology Inc., Danvers, MA, USA) followed by Donkey anti-rabbit Alexa Fluor647 (Biolegend) and analysed by a Zeiss Live Cell Observer microscope. The islet size was examined by compiling measurements of 30 insulin-stained pancreas islets using ImageJ and Zeiss Zen Blue software. Islet macrophages were stained with rabbit anti-Iba1 antibody (dilution 1:400, FUJIFILM Wako Chemicals, Richmond, VA, USA) followed by donkey anti-rabbit Alexa Fluor647 (dilution 1:300, Biolegend). Their numbers were manually counted from 18-21 islet images taken with the microscope. Three animals for each group were analysed.

### Visualization of YFP^+^ BM-Derived Macrophages Within the Islets

Kit^*MerCreMer*^/R26^*YFP*^ fate-mapping mouse was transcardially perfused with 15 ml 4% paraformaldehyde. The pancreas was excised, immersed in 4% paraformaldehyde overnight, followed by dehydration in 30% sucrose before being embedded in OCT. Eight μm sections were cut and stained with anti-Iba1 antibody followed by donkey anti-rabbit Alexa Fluor647. Images were taken by a Zeiss Live Cell Observer microscope.

### Statistical Analysis

Flow cytometry data were analysed using FlowJo 10 (TreeStar, Ashland, OR, USA). Microscopy data were processed with Zeiss Zen Blue and ImageJ. For comparison between two groups, Student’s *t*-test was used to determine the level of significant differences. One-way ANOVA analysis was performed for multiple comparisons of more than two groups. Data were plotted using Prism 9.1.2 (GraphPad Software, La Jolla, CA, USA).

## Results

### Four Main Pancreatic Tissue Resident-Macrophage Subpopulations

**Figure 1:**
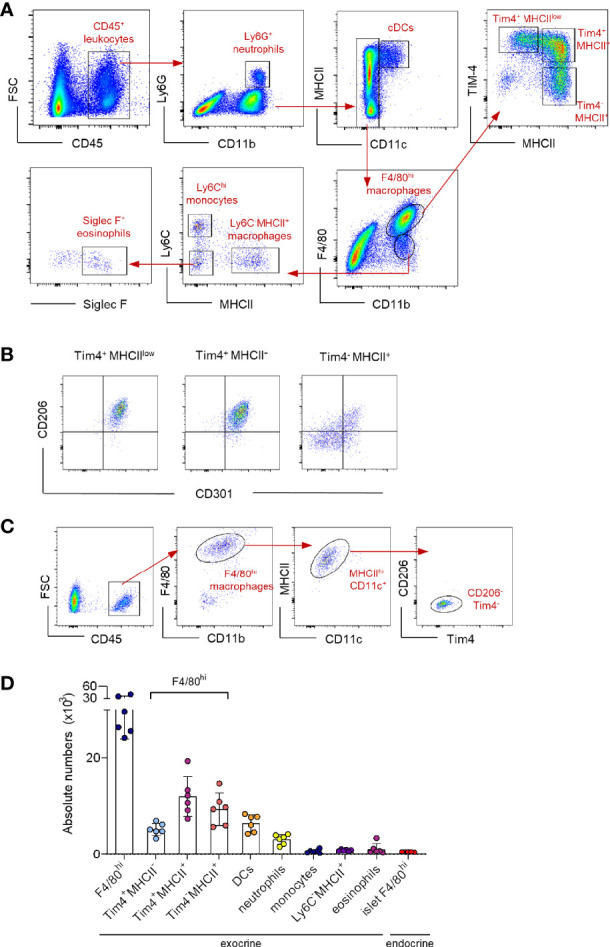
Characterization of the myeloid cell landscape in the pancreas. **(A)** Representative flow cytometry dot plots showing the myeloid cell landscape in the exocrine pancreas of 12-week young mice. Neutrophils: Ly6G; DCs: CD11c^+^MHCII^+^; resident macrophages: F4/80^hi^; resident macrophage subpopulations: Tim4^+^MHCII^low^, Tim4^+^MHCII^+^and Tim4^-^MHCII^+^; monocytes: Ly6C^hi^; monocyte-derived macrophages: F4/80^int^ MHCII^+^; eosinophils: Siglec F^+^. **(B)** Representative flow cytometry dot plots showing the expression profile of CD206 and CD301 in resident intra acinar F4/80^hi^ macrophage subpopulations. **(C)** Representative flow cytometry dot plots showing the islet F4/80^hi^ resident macrophage population in 12-week young mice. Islets obtained from two mice were pooled. **(D)** Bar charts of absolute numbers of distinct myeloid cell populations in exocrine and endocrine pancreas of 12-week young mice. The error bars represent the SD of 6 mice. For the endocrine F4/80^hi^ macrophages, islets were collected from two mice were pooled and error bars represent the SD of 5 pooled samples (total 10 mice).

Pancreatic resident macrophages are present in both endocrine and exocrine areas. The exocrine pancreatic tissue comprises a heterogeneous macrophage family. The F4/80^hi^ tissue-resident macrophages are subdivided into three clear subpopulations based on the expression of embryonic-marker Tim-4 and MHC II (Tim-4^+^MHCII^low^, Tim-4^+^MHCII^+^ and Tim-4^-^MHCII^+^) (**Figure 1A**). Only Tim-4 expressing cells co-express mannose receptor (CD206) and the lectin CD301, whereas the majority of Tim-4^-^ cells do not (**Figure 1B**). In addition, other myeloid cells such as Ly6G^+^ neutrophils, Siglec F^+^ eosinophils, CD11c^+^MHCII^+^ dendritic cells (DCs), Ly6C^+^ monocytes and F4/80^int^Ly6C^-^MHCII^+^ monocyte-derived macrophages are present in the exocrine pancreas, although all in lower frequency (**Figure 1D**). In the islets, according to previously published work (12), the macrophages are the main CD45^+^ cell fraction (> 90%). They represent a small homogenous population expressing F4/80, CD11b, MHC II and CD11c (**Figure 1C**), but negative for Tim-4 and CD206.

In summary, resident macrophages represent the major myeloid population in the pancreas with four distinct subpopulations; a homogenous islet-resident F4/80^hi^ population and a more heterogeneous exocrine-resident F4/80^hi^ cell fraction, consisting of three distinct subpopulations based on the expression of Tim-4 and MHC II.

### Distinct Exocrine Pancreatic Macrophages Differ in Their Turnover Kinetics

**Figure 2:**
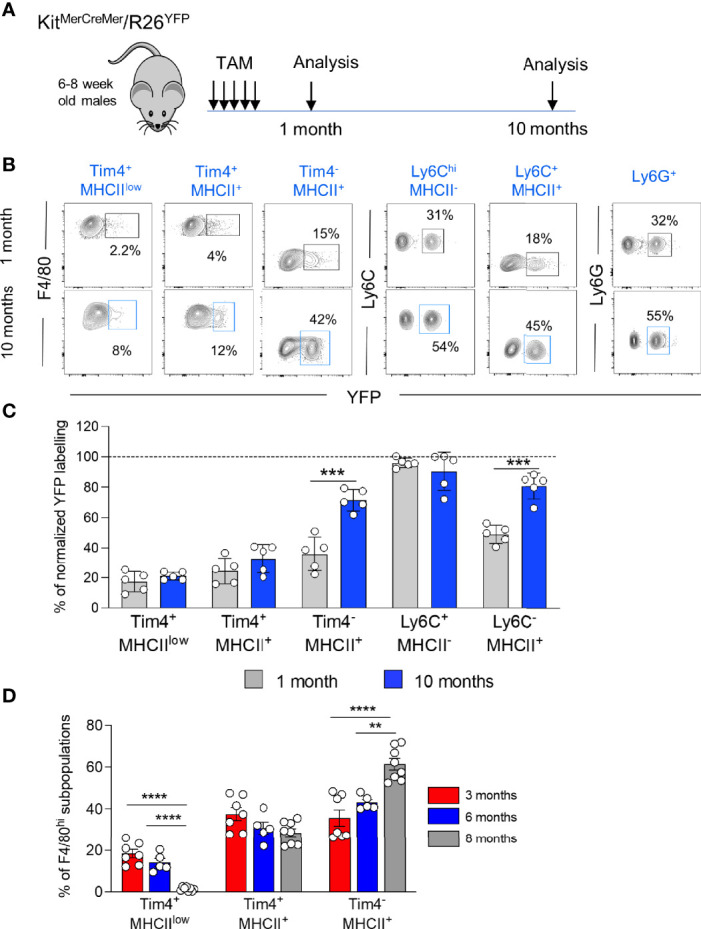
Diverse turnover kinetics within distinct resident macrophages within the exocrine pancreatic tissue. **(A)** Schematic representation of the adult fate-mapping protocol. Male Kit*^MerCreMer^*/R26*^YFP^* mice aged 6-8-weeks were given one dose of tamoxifen per day for five consecutive days and mice from each group (n = 5 mice per group) were sacrificed 1 or 10 months after the last tamoxifen injection. **(B)** Representative flow cytometry contour plots indicating the YFP-labelling of different myeloid cell populations in the exocrine pancreas. **(C)** Bar charts with individual data points showing the percentage of YFP^+^ pancreatic resident exocrine F4/80^hi^ macrophages (Tim4^+^MHCII^low^, Tim4^+^MHCII^+^ and Tim4^-^MHCII^+^), Ly6C^hi^ monocytes and monocyte-derived MHCII^+^ macrophages after normalization to the percentage of YFP^+^ neutrophils of each corresponding mouse. Grey bars: 1 month; blue bars: 10 months. Data of the exocrine cells represent the mean ± SD (n = 5 mice). ***P < 0.001; two-tailed Student’s *t*-test. For clarity, ns values are not shown. **(D)** Bar chart showing the percentage of F4/80^hi^ macrophage subpopulations (Tim4^+^MHCII^low^, Tim4^+^MHCII^+^ and Tim4^-^MHCII^+^) in aging (3, 6 and 8 months). Data represent the mean ± SD of 7 mice (3 month-old mice), 5 mice (6 month-old mice) and 8 mice (8 month-old mice), respectively. **P < 0.01; ****P < 0.0001. One-way ANOVA analysis. For clarity, ns values are not shown.

We used the Kit^*MerCreMer*^/R26^*YFP*^ fate-mapping mouse (9) - where YFP expression can be induced in early c-kit^+^ BM progenitors to investigate the cell replenishment kinetics driven by the BM input in distinct pancreatic myeloid cells (**Figure 2A**). After treating adult Kit^*MerCreMer*^/R26^*YFP*^ mice with tamoxifen (TAM), the YFP labelling index of exocrine tissue-resident macrophages (Tim-4^+^MHCII^low^, Tim-4^+^MHCII^+^ and Tim-4^-^MHCII^+^), monocytes (Ly6C^hi^MHCII^-^), monocyte-derived macrophages (Ly6C^-^MHCII^+^) and neutrophils (Ly6G^+^) was measured by flow cytometry analysis 1 month and 10 months post-TAM gavage. As observed already in other organs (9), neutrophils showed the maximal level of YFP labelling (**Figure 2B**), consistent with their known short half-life and therefore fast turnover rates. Hence for standardization purposes, all other obtained YFP values were normalized to the level of neutrophil labelling, which is considered 100%.

In the exocrine pancreas, as expected, due to their fast-turnover rates, Ly6C^hi^ monocytes and Ly6G^+^ neutrophils showed the highest YFP labelling (**Figures 2B, C**). Resident Tim-4^-^MHCII^+^ and monocyte-derived Ly6C^-^MHCII^+^ macrophages were also gradually replenished by BM-derived cells but at a slower rate. After 10 months of chase, more than 70% of Tim-4^-^MHCII^+^ macrophages and 80% of Ly6C^-^MHCII^+^ macrophages were substituted by monocyte-derived counterparts (**Figure 2C**). Conversely, the remaining two F4/80^hi^ resident exocrine macrophages exhibit minimal YFP labelling (around 20-25%) with most cells remaining unlabeled, hence maintaining their embryonic origin. The Tim-4^+^MHCII^low^ fraction stayed stable at around 20% YFP labelling whereas Tim-4^+^MHCII^+^ macrophages increased by 5% reaching around 30% of YFP^+^ cells (**Figure 2C**). Correlating with the increased refilling kinetics, the Tim-4^-^MHCII^+^ cell fraction within the F4/80^hi^ macrophages progressively increases with age and represents the largest F4/80^hi^ fraction of the pancreatic stroma in older mice, whereas Tim-4^+^MHCII^low^ frequency drastically declines with age (**Figure 2D**).

In summary, the three exocrine subpopulations dynamically change in proportion during aging.

Two stromal Tim-4^+^ resident macrophage subpopulations are long-lived, and most of their cells retain their fetal origin with aging. On the other hand, BM-derived exocrine Tim-4^-^MHCII^+^ resident macrophages are progressively increasing and replacing their embryonic derived counterparts over time.

### Progressive Replacement of Embryo-Derived Islet Macrophages With Aging

**Figure 3:**
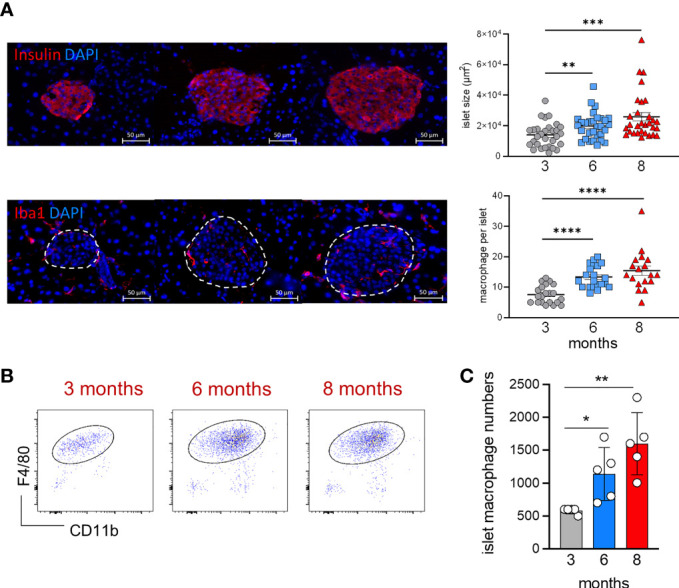
Age‐associated changes in islet size and macrophage numbers. **(A)** Visualization of islet size and islet macrophages in 3, 6 and 8 month-old mice (left). Immunofluorescent staining of insulin [upper panels] and Iba1^+^ macrophages [lower panels] in pancreatic frozen sections. Upper panels, insulin: red; DAPI: blue. Lower panels, Iba1: red; DAPI: blue. Scale bar: 50μm. Charts of islet size and macrophage numbers (right) obtained from frozen sections. 30 islets per group were taken for size measurements, 18-21 islets per group were taken for macrophage numbers. **P < 0.01; ***P < 0.001; ****P < 0.0001. One-way ANOVA analysis. For clarity, ns values are not shown. **(B)** Representative flow cytometry dot plots showing the islet F4/80^hi^ resident macrophage population in 3, 6 and 8 month old mice. Islets obtained from two mice were pooled. **(C)** Bar charts of absolute numbers of islet macrophages obtained from 3, 6 and 8 month old mice. For the endocrine F4/80^hi^ macrophages, islets were obtained from two mice were pooled and error bars represent the SD of 5 pooled samples (total 10 mice). *P < 0.05; **P < 0.01. One-way ANOVA analysis. For clarity, ns values are not shown.

**Figure 4:**
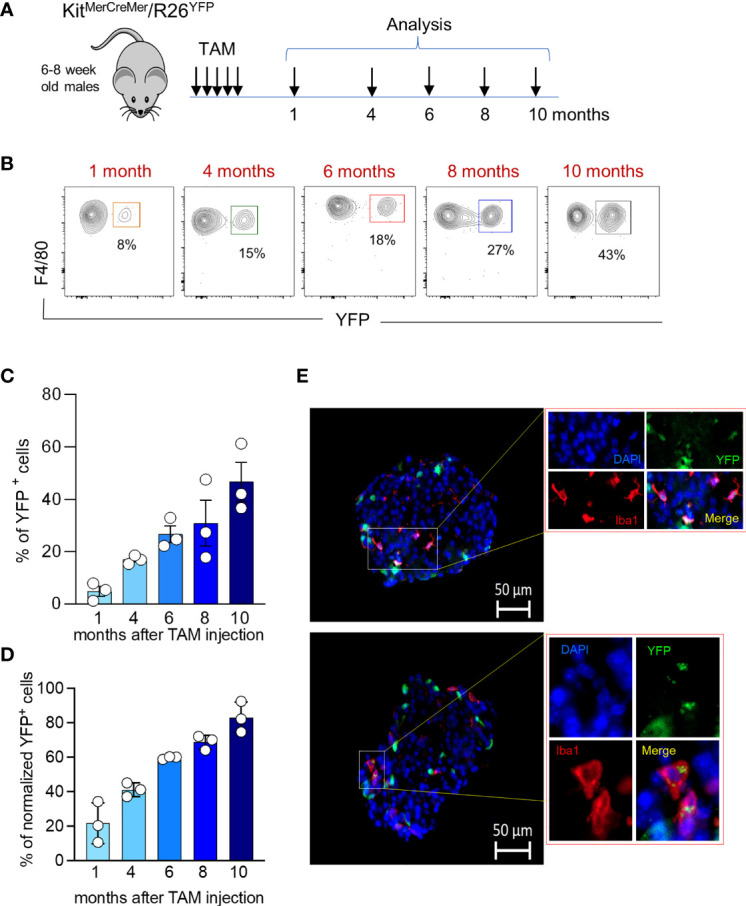
Age‐associated changes in islet macrophage turnover kinetics. **(A)** Schematic representation of the adult fate-mapping protocol. Male Kit*^MerCreMer^*/R26*^YFP^* mice aged 6-8 weeks were given one dose of tamoxifen per day for five consecutive days and mice from each group (n= 6 mice per group) were sacrificed 1, 4, 6, 8 or 10 months after the last tamoxifen injection. Islets obtained from two mice were pooled. **(B)** Representative flow cytometry contour plots indicating the YFP-labelling of islet macrophages at distinct time points (1, 4, 6, 8 or 10 months post TAM treatment). **(C)** Bar charts with individual data points showing the percentage of YFP^+^ pancreatic resident endocrine F4/80^hi^ macrophages before normalization. Error bars represent the SD of 3 pooled samples (total 6 mice). **(D)** Bar charts with individual data points showing the percentage of YFP^+^ pancreatic resident endocrine F4/80^hi^ macrophages after normalization to the mean percentage of YFP^+^ neutrophils obtained from the same 2 mice. Error bars represent the SD of 3 pooled samples (total 6 mice). **(E)** Visualization of YFP^+^ Iba1^+^ cells within the islets. Immunofluorescent staining of Iba1^+^ macrophages in pancreatic frozen sections. The pancreas was collected from 7 months old mice which were labelled for 6 months. Iba1: red; YFP: green; DAPI: blue. Scale bar: 50μm.

During aging, islet size and islet macrophage numbers (**Figures 3A–C**) significantly increase over time (**Figures 3A–C**). We performed an extensive long-term fate-mapping analysis, to investigate whether BM-derived cells contribute to replenishing the embryonic-derived islet macrophages. The YFP labelling index of endocrine tissue-resident macrophages was measured in Kit^*MerCreMer*^/R26^*YFP*^ mice at several time points (1, 4, 6, 8 and 10 months) post-TAM gavage (**Figure 4A**). Each sample of islets was pooled from two mice, and the normalization was done using the mean neutrophil YFP percentage of the same two mice. In the pancreatic islets, resident F4/80^hi^ macrophages were gradually replaced over time by BM-derived cells (**Figures 4B, C**), reaching after 10-month TAM treatment around 70% of normalized YFP labelling (**Figure 4D**). To validate our flow cytometry results and to visualize the YFP^+^ macrophages directly within the islet, we performed immunofluorescent staining using Iba-1 as a surrogate marker for resident islet macrophages (22). Our analysis confirms the presence of YFP^+^Iba1^+^ cells at the capsular and in the intra-islet region (**Figure 4E**) which confirms that embryonic-derived islet macrophages are replenished by BM-derived cells over time.

### The Pancreas Harbours Both CCR2-Dependent and CCR2-Independent Resident Macrophages

**Figure 5:**
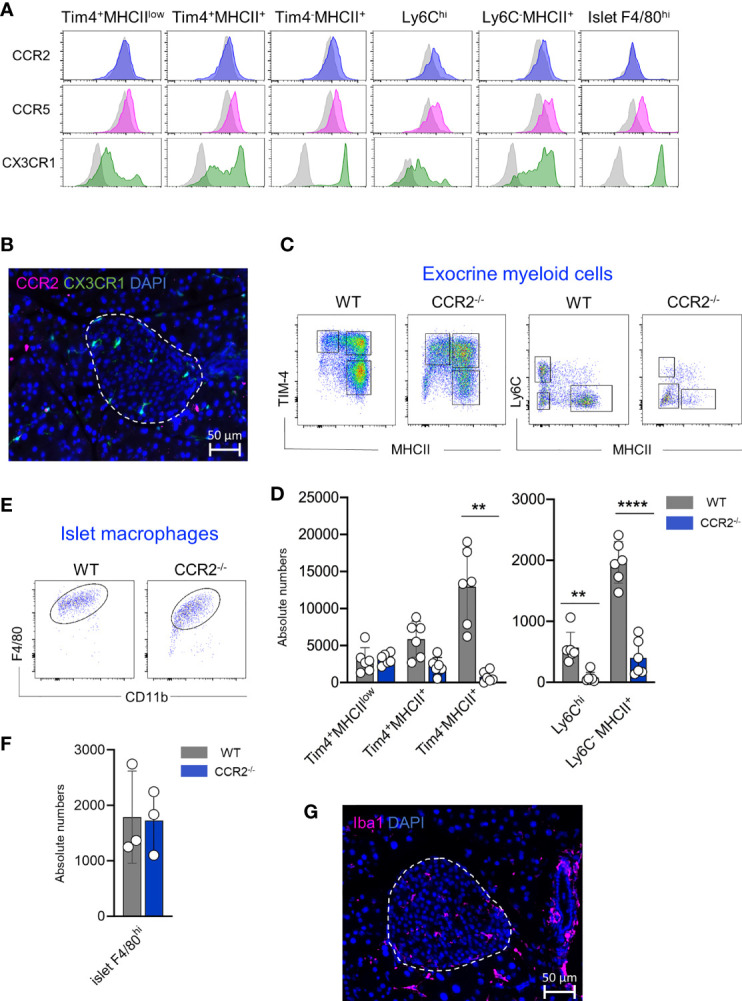
Pancreatic resident macrophage subpopulations exhibit diverse CCR2 dependency. **(A)** CCR2, CCR5 and CX3CR1 expression profile of exocrine resident F4/80^hi^ macrophages (Tim4^+^MHCII^low^, Tim4^+^MHCII^+^ and Tim4^-^MHCII^+^), Ly6C^hi^ monocytes and monocyte-derived MHCII^+^ macrophages and islet resident F4/80^hi^ resident macrophages. Light grey histograms show correspondent negative control. **(B)** Representative visualization of CCR2 expressing cells within the islet obtained from ND CX3CR1^GFP^ mice. CCR2: pink; CX3CR1: green; DAPI: blue. Scale bar: 50μm. **(C)** Flow cytometry representative dot plots showing exocrine F4/80^hi^ macrophage (left) and F4/80^int^ Ly6C^hi^ monocytes and F4/80^int^ MHCII^+^ macrophage distribution in WD WT and CCR2^-/-^ mice. **(D)** Bar charts with individual data points showing correspondent absolute cell numbers of exocrine F4/80^hi^ macrophage and F4/80^int^ Ly6C^hi^ monocytes and F4/80^int^ MHCII^+^ macrophage of 6 independent WD WT and CCR2^-/-^ mice. **P < 0.01; ****P < 0.0001; two-tailed Student’s *t*-test. For clarity, ns values are not shown. **(E)** Flow cytometry representative dot plots showing endocrine F4/80^hi^ macrophages in WD WT and CCR2^-/-^ mice. **(F)** Bar charts with individual data points showing correspondent absolute cell numbers of endocrine F4/80^hi^ macrophages. Islets obtained from two mice were pooled and error bars represent the SD of 3 pooled samples (total 6 mice). The error bars represent the SD. Two-tailed Student’s *t*-test. For clarity, ns values are not shown. **(G)** Immunofluorescent staining of Iba1^+^ macrophages in pancreatic frozen sections. Obtained from WD CCR2^-/-^ mice. Iba1: pink; DAPI: blue. Scale bar: 50μm.

Chemokine/chemokine receptor interactions control the migration of immune cells. Chemokine receptors such as CCR2, CCR5 and CX3CR1 regulate the recruitment of the monocytes into the tissues (23–25). Therefore, we analyzed the expression of these three main chemokine receptors on monocytes, monocyte-derived macrophages, resident macrophages isolated from the exocrine region and macrophages obtained from the islets. In the exocrine pancreas, only Ly6C^hi^ monocytes, monocyte-dependent F4/80^int^ macrophages and F4/80^hi^Tim-4^-^MHCII^+^ macrophages expressed CCR2 on the surface of their cells (**Figure 5A**) whereas Tim-4^+^MHCII^low^ and Tim-4^+^MHCII^+^ macrophages showed minimal CCR2 expression. CCR5 was expressed similarly on all tested myeloid cells, whereas high expression of CX3CR1 was restricted to F4/80^hi^Tim-4^-^MHCII^+^ macrophages and monocyte-derived F4/80^int^ macrophages. Interestingly, the expression of CCR2 on islet F4/80^hi^ macrophages was weak or almost undetectable with the antibody used in this study, whereas CCR5 and CX3CR1 were clearly expressed on their cell surface (**Figure 5A**). Immunofluorescence staining also confirmed that no CCR2^+^ cells were detected in the islet, whereas clearly, CX3CR1^+^ cells were visible within the endocrine area (**Figure 5B**).

Since the CCL2/CCR2 axis refills the resident macrophage pool in tissues such as intestine, adipose tissue, heart, and skin, we investigated the contribution of this axis to the replenishment of tissue-resident macrophages in the pancreas. We analysed their frequency and absolute numbers in exocrine and endocrine pancreas of WT and CCR2^-/-^ mice, the latter mouse strain known for its impaired egress of Ly6C^hi^ monocytes from the BM and lack of peripheral monocytes (26). Both mouse strains were fed with WD for 24 weeks and were analyzed at an age of 8 months. Correlating with the CCR2 expression, we found that intra acinar Ly6C^hi^ monocytes, monocyte-derived F4/80^int^MHCII^+^ macrophages and F4/80^hi^Tim-4^-^MHCII^+^ macrophage numbers were significantly reduced in CCR2^-/-^ mice when compared to WT counterparts. Conversely, the numbers of Tim-4^+^MHCII^low^ and Tim-4^+^MHCII^+^ macrophage subsets were similar in WT and CCR2^-/-^ mice (**Figures 5C, D**). Interestingly, islet F4/80^hi^ macrophages were clearly detectable in both mouse strains and their numbers were comparable (**Figures 5E, F**). Their presence within the islets was further validated by immunohistochemistry staining on pancreas sections obtained from CCR2^-/-^ mice (**Figure 5G**).

In summary, the maintenance of long-lived exocrine TIM-4^+^ macrophages is CCR2 independent. Surprisingly, islet resident F4/80^hi^ macrophage numbers are not reduced in CCR2^-/-^ mice, hence their replenishment is independent of the CCL2/CCR2 axis and modulated possibly by other chemokine receptors, such as CCR5 and CX3CR1.

### Obesity and Diabetes Do Not Accelerate Macrophage Turnover Kinetics

**Figure 6:**
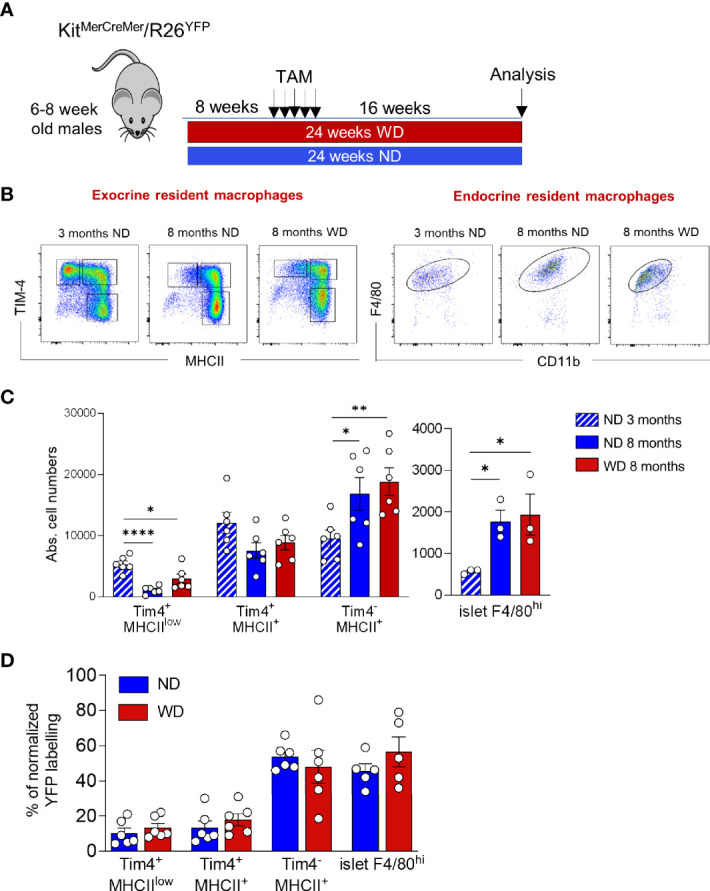
Obesity and T2DM do not accelerate macrophage turnover kinetics. **(A)** Schematic representation of the adult fate-mapping protocol in lean and obese mice. Male Kit*^MerCreMer^*/R26*^YFP^* mice aged 6-8-weeks were fed with ND or WD over a 24-week period. Sixteen weeks before the euthanasia, mice were given five consecutive daily doses of tamoxifen. **(B)** Flow cytometry representative dot plots showing distinct pancreatic F4/80^hi^ resident subpopulations in young ND-fed mice (3-month old), in aged ND-fed mice (8-month old) and in aged WD fed mice obese (8-month old). Left: exocrine pancreas: *x-*axis: MHCII and *y*-axis: TIM4. Right: endocrine pancreas: *x-*axis: CD11b and *y*-axis: F4/80. **(C)**. Absolute numbers of resident macrophage subpopulations in exocrine (left panel) and endocrine pancreas (right panel) in young (3-months old), aged and obese mice (8-months old). The error bars represent the SD of 6 mice. For the endocrine F4/80^hi^ macrophages obtained from two mice were pooled and error bars represent the SD of 3 pooled samples (total 6 mice). *P < 0.05; **P < 0.01; ****P < 0.0001; two-tailed Student’s *t*-test. For clarity ns values are not shown. **(D)** Bar charts with individual data points showing the percentage of YFP^+^ pancreatic resident exocrine F4/80^hi^ macrophages (Tim4^+^MHCII^low^, Tim4^+^MHCII^+^ and Tim4^-^MHCII^+^) and islet F4/80^hi^ macrophages after normalization to the percentage of YFP^+^ neutrophils. Blue bars: ND; red bars: WD. For the exocrine error bars represent the SD of 6 mice. For the islets error bars represent the SD of 5 pooled samples (total 10 mice). Two-tailed Student’s *t*-test. For clarity, ns values are not shown.

We have recently shown that obesity accelerates the turnover kinetics of adipose tissue-resident macrophages (27). To assess whether obesity affects the replenishment of distinct pancreatic macrophage subpopulations, we established another adult fate-mapping experiment in Kit^*MerCreMer*^/R26^*YFP*^ mice maintained either on a normal chow diet (NCD) or on a western diet (WD) for 24 consecutive weeks (**Figure 6A**). When compared to 3 months old mice, both experimental groups increased their body weights during aging, while the WD-fed mice were overweight and diabetic (**Table 1**). In fact, mean body weight and fasting blood glucose were significantly higher in WD-fed mice compared with aged-matched NCD-fed controls. The islet area also increased with aging but was equal between the two aged-matched NCD- and WD-ed groups (**Table 1**). Exocrine and endocrine resident macrophage subpopulations were subsequently analyzed by flow cytometry and YFP labelling was monitored as a parameter for the monocyte-dependent replenishment. As already observed in aging mice (**Figure 2D**), the exocrine Tim-4^-^MHCII^+^ cells represent the largest F4/80^hi^ fraction of the pancreatic stroma in obese mice, whereas both Tim-4^+^ populations decrease their frequency and numbers (**Figures 6B, C**, left panels). In WD-fed mice, islet macrophage numbers were increased when compared to young lean mice but were comparable with aged lean (**Table 1**, **Figures 6B, C**, right panels). Although it was reported that obesity affects the replenishment kinetics of adipose tissue-resident macrophages (23), in the pancreas, there was no significant perturbation. Both Tim-4^+^ subpopulations remained minimally labeled and maintained their long-lived phenotype also in the tissue microenvironment of obese mice (**Figure 6D**). Tim-4^-^MHCII^+^ and islet macrophages were also comparably YFP labeled between lean and obese aged mice (**Figure 6D**), which indicates that obesity did not accelerate the monocyte driven replacement and resident macrophages maintained the same turnover kinetics.

**Table 1:** WD-fed mice become diabetic by 8 months of age.

	3 months (NCD)	8 months (NCD)	8 months (WD)
Body weight (g)^a^	21.13 +/- 1	34.9 +/- 5	45.8 +/- 2.8
Glucose (mmol/L)^b^	5.65 +/- 0.83	6.05 +/- 1.5	11.77 +/- 1.3
Insulin (μg/L)^c^	0.27+/-0.14	0.40+/-0.26	1.98+/-0.86
Size of islets (μm^2^)^d^	13,922 +/- 1,473	25,957+/- 2,777	33,213 +/- 4,005
Numbers of F4/80^hi^ macrophages/islet^e^	7.51 +/- 0.64	15.44 +/- 1.53	15.75 +/- 1.13

a Body weight data are presented as mean values +/- SD of 6 male mice. Statistical significance was assessed by one-way ANOVA. 8 months NCD vs 3 months NCD mouse group: ****P < 0.0001; 8 months WD vs 3 months NCD mouse group: ****P < 0.0001; 8 months NCD vs 8 months WD: ***P < 0.001.

b Fasting glucose data are presented as mean values +/- SD of 6 male mice. Statistical significance was assessed by one-way ANOVA. 8 months NCD vs 3 months NCD mouse group: ns; 8 months WD vs 3 months NCD mouse group: ****P < 0.0001; 8 months NCD vs 8 months WD: ****P <0.0001.

c Fasting insulin data are presented as mean values +/- SD of 6 male mice. Statistical significance was assessed by one-way ANOVA. 8 months NCD vs 3 months NCD mouse group: ns; 8 months WD vs 3 months NCD mouse group: ***P < 0.001; 8 months NCD vs 8 months WD: ***P <0.001.

d The mean +/- SEM of the islet size is calculated from 30 islets/group. Statistical significance was assessed by one-way ANOVA; 8 months NCD vs 3 months NCD mouse group: *P < 0.05; 8 months WD vs 3 months NCD mouse group: ****P < 0.0001; 8 months NCD vs 8 months WD: ns.

e The mean +/- SEM of F4/80hi macrophages/islet from 18-21 islets. Statistical significance was assessed by one-way ANOVA. 8 months NCD vs 3 months NCD mouse group: ****P < 0.0001; 8 months WD vs 3 months NCD mouse group: ****P < 0.0001; 8 months NCD vs 8 months WD: ns.

## Discussion

Here we have studied pancreatic resident macrophages and their relative frequencies, monocyte-dependent replenishment and turnover kinetics in aging under normal homeostasis and obese diabetic conditions.

Although pancreatic macrophages have already been identified and described in previous studies (12–14, 17, 28–31), our data bring additional new insights regarding their phenotype and replenishment kinetics. We have identified four distinct resident F4/80^hi^ pancreatic macrophages; three in the exocrine and one in the endocrine tissue. The CD206^+^CD301^+^ stromal macrophages identified by Calderon et al. in 2015 (12), could be further separated into two distinct subpopulations by the expression of the embryonic marker Tim-4 and MHC II. Both markers have been used as valuable tools for the characterization of long-lived intestinal (32), heart (33), and adipose tissue macrophages (27). The majority of the remaining inter acinar F4/80^hi^ macrophages lack the expression of CD206, CD301 and Tim-4 but express MHC II. Interestingly, aging affects the frequency of these three macrophages within the stromal microenvironment, with the Tim-4-MHCII^+^ population exceeding the Tim-4^+^ fractions over time and becoming the dominant fraction in older healthy and obese mice. In alignment with previous work (12), islet F4/80^hi^ macrophages, when isolated and analysed by flow cytometry, display a homogenous macrophage population expressing CD11c and MHC II and represent the main leukocyte fraction in the exocrine tissue. Our flow cytometry analysis could not further separate the exocrine macrophages into CD11c^+^ intra- and CD11c^-^ peri-islet macrophages as recently described by Ying et al. (31) in young nor in aged or obese mice.

Similarly to other organs, turnover rates of macrophages in the pancreas vary between the different resident macrophage subpopulations. The stromal compartment contains two long-lived resident Tim-4^+^ macrophage subpopulations that minimally interchanged with BM-derived cells and maintained their embryonic signature during aging. Similar long-lived Tim-4^+^ macrophages with slow replenishment kinetics have been observed in adipose tissue (27), intestine (32, 34) as well as in the heart (33). On the contrary, the third Tim-4^-^MHCII^+^ exocrine subset is gradually replaced by BM-derived monocytes and loses its embryonic origin with the progression of aging. Unlike the Tim-4^+^ macrophages, the Tim-4^-^MHCII^+^ fraction expresses the CCR2 chemokine receptor and is almost totally absent in CCR2^-/-^ mice lacking circulating monocytes. Thus, only this F4/80^hi^ macrophage subpopulation requires monocyte replenishment for its maintenance in the exocrine microenvironment. Our results are in agreement with past studies, although the previous analysis was limited only to two CD206^+^CD301^+^ and CD206^-^CD301^-^ macrophage stromal fractions (12). Interestingly, obesity does not affect the ratio between the three exocrine resident macrophages, nor does it accelerate their turnover rates, as was described in the adipose tissue (27, 35).

Endocrine F4/80^hi^ macrophages have been described as long-lived embryonic-derived cells that self-renew locally within the islets since birth. Parabiosis experiments showed that host-derived monocytes hardly replace islet macrophages (12). Furthermore, adoptively transferred isolated monocytes were almost exclusively restricted to the peri-islet capsule area and did not penetrate into the intra-islet compartment nor differentiate into islet macrophages (31). Although these two previous studies evidenced the monocyte-independency of islet macrophages, our fate mapping results clearly indicate that endocrine resident macrophages are gradually replaced by monocytes during aging. The difference between these two opposite results could rely on the timeline used during the experiments. Our fate mapping analysis monitored a time window of 10 months, therefore, captured the refilling event during a longer period of time. In comparison, joined mice were analysed after 6 weeks of parabiosis, hence the turnover kinetic measurement was limited to a short window. In accordance with the parabiosis experiments, at a similar early time point (e.g. 1 month post-TAM injected) our adult fate-mapping data revealed comparable low YFP F4/80^hi^ macrophage labelling. However, 8 months later, the majority of embryonic-derived islet macrophages were gradually replaced by BM-derived monocytes. Thus, in aging mice, embryonic-derived and postnatal BM-derived F4/80^hi^ macrophages coexist in the islet microenvironment due to the gradual replacement of the embryonic-derived macrophages by the monocyte-derived ones. Surprisingly the contribution of BM-derived monocytes to the replenishment of the endocrine macrophage population was CCR2 independent since islet macrophage numbers were unaffected in both chemokine receptor-deficient mice. Possibly other chemokines such as CCR5 or CX3CR1, highly expressed on endocrine macrophages, could mediate the replenishment kinetics in the islets (23).

Recent studies have suggested that obesity can drive inflammation and metabolic changes within the pancreatic microenvironment (31), raising the possibility that obesity accelerates the macrophage turnover kinetics in overweight mice as observed in the adipose tissue (27). It has been reported that obese and T2DM individuals and animals accumulate augmented macrophage numbers in the islets (29, 36). A significant pancreatic islet size increase was reported in the first four months of high-fat diet treatment when compared to aged-matched NCD-fed mice (21, 37). However, this diet-mediated difference in islet size disappeared in twelve-month-old lean and obese mice (37), consistent with what we have observed in 8 month-old lean and obese mice showing a comparable islet size expansion and an equivalent amount of islet macrophages. Moreover, 24-week-long WD treatment of mice did not accelerate the replenishment of the embryonic-derived macrophages, neither in the endocrine nor in the exocrine compartment, which indicates that WD-driven obesity and T2DM does not influence the pancreatic macrophage turnover rates.

In conclusion, we propose, in contrast to the current dogma (12), that age progression allows the gradual replacement of embryonic-derived islet macrophages by adult BM-derived macrophages, and we provide novel evidence that the replenishment happens independently from the conventional CCL2/CCR2 axis. Furthermore, our fate-mapping analysis reveals that obesity does not accelerate turnover kinetics of any endocrine and exocrine pancreatic macrophage subpopulation as it was observed in the adipose tissue, bringing some unexpected new insights into the biology of pancreatic macrophages.

## Data Availability Statement

The datasets presented in this study can be found in online repositories. The names of the repository/repositories and accession number(s) can be found below: The original flow cytometry data have been deposited in the NTU Open Access Data Repository (DR-NTU) at https://doi.org/10.21979/N9/2YJP6X.

## Ethics Statement

The animal studies involving mice were reviewed and approved by the Institutional Animal Care and Use Committee of the Nanyang Technological University (ARF- SBS/NIE A18081 and 19093).

## Author Contributions

Conceptualization, CR. Methodology, ZM. Formal analysis, ZM and CR. Writing, CR. Visualization, ZM and CR. Supervision, CR. Funding acquisition, CR. All authors contributed to the article and approved the submitted version.

## Funding

Funding for this paper was provided by a Ministry of Education Tier2 grant (MOE2018-T2-2-016) awarded to CR.

## Conflict of Interest

The authors declare that the research was conducted in the absence of any commercial or financial relationships that could be construed as a potential conflict of interest.

## Publisher’s Note

All claims expressed in this article are solely those of the authors and do not necessarily represent those of their affiliated organizations, or those of the publisher, the editors and the reviewers. Any product that may be evaluated in this article, or claim that may be made by its manufacturer, is not guaranteed or endorsed by the publisher.
